# The natural sulfoglycolipid derivative SQAP improves the therapeutic efficacy of tissue factor-targeted radioimmunotherapy in the stroma-rich pancreatic cancer model BxPC-3

**DOI:** 10.1016/j.tranon.2021.101285

**Published:** 2021-11-25

**Authors:** Yoichi Takakusagi, Aya Sugyo, Atsushi B. Tsuji, Hitomi Sudo, Masahiro Yasunaga, Yasuhiro Matsumura, Fumio Sugawara, Kengo Sakaguchi, Tatsuya Higashi

**Affiliations:** a Department of Molecular Imaging and Theranostics, Institute for Quantum Medical Science, National Institutes for Quantum and Radiological Science and Technology (QST-iQMS), 4-9-1 Inage, Chiba 263-8555, Japan; b Institute for Quantum Life Science, National Institutes for Quantum and Radiological Science and Technology (QST-iQLS), 4-9-1 Inage, Chiba 263-8555, Japan; c Division of Developmental Therapeutics, Exploratory Oncology Research and Clinical Trial Center, National Cancer Center, 6-5-1 Kashiwanoha, Kashiwa, Chiba 277-8577, Japan; d Department of Immune Medicine, National Cancer Center Research Institute 6-5-1 Kashiwanoha, Kashiwa, Chiba 277-8577, Japan; e pplied Biological Science, Faculty of Science and Technology, Tokyo University of Science, 2641 Yamazaki, Noda, Chiba 278-8510, Japan; f Malignant Tumor Treatment Technologies (M.T.3) Inc., 3-20-2 Shibaura, Minato-ku, Tokyo 108-0023, Japan

**Keywords:** Refractory cancer, Molecular radiotherapy, Therapeutic nuclear medicine, Radionuclide, Neoangiogenesis

## Abstract

α-Sulfoquinovosylacyl-1,3-propanediol (SQAP) is a semi-synthetic derivative of natural sulfoglycolipid that sensitizes tumors to external-beam radiotherapy. How SQAP affects internal radiotherapy, however, is not known. Here, we investigated the effects of SQAP for radioimmunotherapy (RIT) targeting tissue factor (TF) in a stroma-rich refractory pancreatic cancer mouse model, BxPC-3. A low dose of SQAP (2 mg/kg) increased tumor uptake of the ^111^In-labeled anti-TF antibody 1849, indicating increased tumor perfusion. The addition of SQAP enhanced the growth-inhibitory effect of ^90^Y-labeled 1849 without leading to severe body weight changes, allowing for the dose of ^90^Y-labeled 1849 to be reduced to half that when used alone. Histologic analysis revealed few necrotic and apoptotic cells, but Ki-67–positive proliferating cells and increased vascular formation were detected. These results suggest that the addition of a low dose of SQAP may improve the therapeutic efficacy of TF-targeted RIT by increasing tumor perfusion, even for stroma-rich refractory pancreatic cancer.

## Highlights

• SQAP enhanced tumor uptake and the therapeutic efficacy of radiolabeled anti-tissue factor antibody 1849.
• SQAP allows for a reduction of the dose of the therapeutic agent ^90^Y-labeled 1849 to half.
• SQAP did not affect hematologic parameters, or gastrointestinal and respiratory systems in mice.
• ^90^Y-labeled 1849 with SQAP potentially increases exposure of tumors to radiation.

## Graphical abstract

**Figure:**
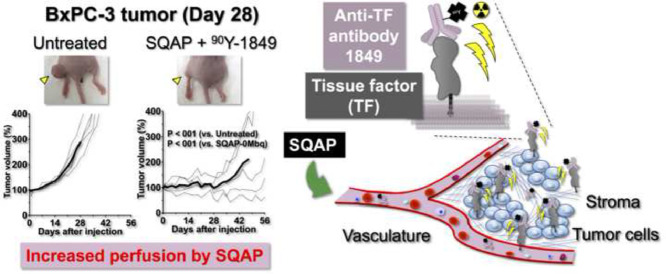

## Introduction

Pancreatic cancer is a highly lethal cancer with a 5-year survival rate of 10% for all stages of the disease [1]. It is projected to be the second leading cause of cancer death in the United States by 2030 [2]. The disease progresses asymptomatically in 80% of patients and is thus usually detected in an advanced stage with local invasion and/or metastasis, resulting in unresectable cancer [3]. Among the 10%–15% of patients who present with resectable disease, 80% experience a relapse [4]. Various chemotherapeutic agents are applied for locally advanced and metastatic disease, but with limited outcomes [5]. Therefore, new systemic treatment strategies are needed.

A malignant cycle of blood coagulation is postulated to generate versatile cancer stroma, leading to cancer invasion into vessels, tumor proliferation, and replacement with collagenous tissue [6]. Cancer coagulopathy is triggered by tissue factor (TF), which is a transmembrane glycoprotein (47 kDa) present on the cell surface [7]. Abnormally high TF expression is found in various tumors, including pancreatic cancer [8], [9], [10]. High TF expression in pancreatic cancer correlates with tumor grade, extent, metastasis, and invasion, in contrast to normal pancreas with low TF expression [7, 9, 11]. TF is expressed not only on the tumor cell surface but also in the tumor stroma and on tumor-associated vascular endothelial cells [8, 12]. Therefore, TF is a potential target for cancer diagnostic imaging and therapy for pancreatic cancer with a rich stroma.

We developed high-affinity anti-TF antibodies and demonstrated that radiolabeled anti-TF monoclonal antibody 1849 has high potential as a noninvasive imaging probe [13]. In glioma and pancreatic cancer xenograft models, ^111^In-labeled 1849 exhibits high uptake in tumors and low uptake in normal organs [14, 15]. Radiolabeled 1849 in which the imaging radionuclide ^111^In was replaced with a β-emitting therapeutic radionuclide, such as ^90^Y and ^177^Lu, is a promising radioimmunotherapy (RIT) agent for metastatic pancreatic cancer. The pancreatic cancer model BxPC-3, however, exhibits resistance to monotherapy with ^90^Y-labeled antibodies [16], [17], [18]. RIT with ^90^Y-labeled antibodies achieved complete remission in a stroma-poor model, MIA-PaCa-2, whereas the stroma-rich pancreatic cancer model BxPC-3 showed a limited efficacy of progressive disease [14, [19], [20], [21], [22], [23]]. The rich stroma is involved in the resistance, which contributes to the inhibition of antibody penetration as the main barrier for RIT [23]. BxPC-3 shows the most radioresistant to RIT with ^90^Y-labeled antibodies [23] and is the only clinically relevant stroma-rich pancreatic cancer model available to our knowledge. Even though the highest level of TF expression on both tumor parenchyma and stroma in BxPC-3, TF-targeted RIT with ^90^Y-labeled antibodies stated only moderate effect [24]. If additional strategies to enhance the therapeutic efficacy of RIT beyond such therapeutic resistance can be established, those would serve as a superior therapeutic option even for the highest class of stroma-rich refractory pancreatic cancer.

**Fig. 1:**
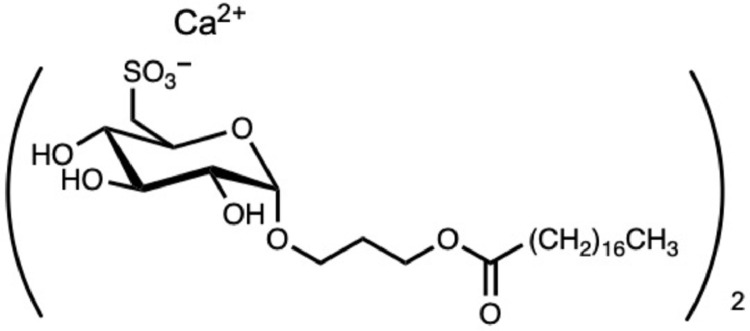
Molecular structure of SQAP.

Sulfoquinovosylacylglycerol (SQAG) is a sulfoglycolipid originally isolated from natural sources, including higher plants [25, 26], sea urchins [27], and marine algae [28]. SQAG has tumor-radiosensitizing properties [29, 30]. A synthetic analog of SQAG, α-sulfoquinovosylacyl-1,3-propanediol (SQAP or CG-0321) (Fig. 1) was synthesized for clinical studies [31] and acts as a radiosensitizer like SQAG [32, 33]. This compound induces pharmacologic alterations of the tumor microenvironment; low-dose (2 mg/kg) intravenous administration of SQAP increases tumor perfusion and oxygenation, thereby enhancing external-beam radiotherapy by boosting the oxygen effect, which significantly delays the growth of murine SCCVII and human A549 xenografts [32]. This finding has attracted attention to SQAP as a potentially useful option for improving the therapeutic efficacy of internal radiotherapy for cancer.

The present study evaluated whether SQAP improves the therapeutic efficacy of RIT in the stroma-rich refractory pancreatic cancer model BxPC-3. An anti-TF monoclonal antibody 1849 labeled with the γ-emitter ^111^In with 171 keV and 245 keV, and a half-life of 67.4 h [34], was produced. The effect of SQAP on the biodistribution of ^111^In-labeled 1849 was assessed. The therapeutic efficacy of the antibody labeled with a β-emitter ^90^Y (maximum energy, 2.3 MeV; half-life, 64.1 h [34]) substituted for ^111^In was examined in combination use with SQAP.

## Materials and methods

### Measurement of the hematologic parameters

SQAP (Toyo Suisan, Tokyo, Japan) (Fig. 1) was dissolved in saline (Otsuka Pharmaceutical, Tokyo, Japan). Male 5-week-old BALB/c-nu/nu mice (*n* = 2; CLEA Japan, Tokyo, Japan) were injected intravenously with SQAP (2, 24, 28, and 32 mg/kg body weight) or saline. Blood was collected *via* the tail vein on day 1 and analyzed with a Celltac Alpha hematology analyzer (Nihon Kohden, Tokyo, Japan). Details are provided in the Supplementary information.

### Radiolabeling of the antibody

The monoclonal antibody 1849 recognizing human TF [13, 35] was conjugated with *p*-SCN-Bn-CHX-A''-DTPA (DTPA; Macrocyclics, Dallas, TX, USA) as previously described [23] (details are provided in the Supplementary information). The labeling yield of ^111^In-1849 was 86.1%, and the labeling yields of ^90^Y-1849 were 87.5% to 90.7%. The radiochemical purity of the labeled antibodies was almost 100%. The specific activity of ^111^In-1849 was 31 kBq/μg, and the specific activity of ^90^Y-1849 was 767 kBq/μg and 829 kBq/μg.

### Cells and tumor-bearing mice

The human pancreatic cancer cell line BxPC-3 (ATCC, Manassas, VA, USA) was maintained in RPMI1640 medium (Wako Pure Chemical Industries, Osaka, Japan) supplemented with 10% fetal bovine serum (Sigma-Aldrich, St. Louis, MO, USA). Male BALB/c-nu/nu mice (5 weeks old) were inoculated subcutaneously with BxPC-3 cells (4 × 10^6^) in the left thigh under isoflurane anesthesia.

### Biodistribution and absorbed dose estimation

The Animal Care and Use Committee of the National Institute of Radiological Sciences approved the protocol for the animal experiments, and all animal experiments were conducted by following the Institutional Guidelines regarding Animal Care and Handling. When subcutaneous tumors reached a diameter of approximately 8 mm, mice (*n* = 5/time-point) were injected intravenously with 37 kBq of ^111^In-labeled 1849. The total injected protein dose was adjusted to 10 µg per mouse by adding intact 1849. Saline and SQAP (2 mg/kg body weight) were administered intravenously 20 min before injection of the radiolabeled antibody, and 1 and 2 days after the injection. At 1, 6, 24, 48, 96, and 168 h after injection of ^111^In-1849, the mice were humanely killed by overexposure to isoflurane. Tumors, and tissues and organs of interest (blood, lung, liver, spleen, pancreas, intestine, kidney, muscle, and bone) were removed and weighed, and radioactivity was measured using a gamma-counter (2470 WIZARD^2^, PerkinElmer, Waltham, MA, USA). The data were expressed as the percentage of injected dose per gram of tissue (% ID/g) normalized to a mouse with a body weight of 20 g. The absorbed dose for ^90^Y-labeled 1849 was estimated using the area under the curve of each organ based on the biodistribution data of ^111^In-1849 and the mean energy emitted per transition of ^90^Y, 1.495 × 10^−13^ Gy kg (Bq s)^−1^ [36] as described previously [37].

### Therapeutic experiments

When subcutaneous tumors reached a diameter of approximately 8 mm, therapeutic experiments were conducted. The mice (*n* = 5/dose) were injected into a tail vein with 0.925, 1.85, or 3.7 MBq of ^90^Y-labeled 1849. The protein dose was adjusted to 10 µg for each preparation by adding intact antibody. Saline and SQAP (2 mg/kg body weight) were administered intravenously 20 min before injection of the radiolabeled antibody, and 1 and 2 days after the injection. Untreated mice (*n* = 5) were used as a control. Body weight and tumor size were measured at least 3 times a week for 8 weeks. When the tumor reached 12 mm in diameter, the mouse was humanely killed by overexposure to isoflurane. Tumor volume (mm^3^) was calculated as (length × width^2^)/2.

### Histologic analysis of tumors

As a separate experiment, tumor samples (*n* = 3/time-point) were extirpated at days 1, 2, and 5 after intravenous injection of intact 1849 (0 MBq) or 3.7 MBq of ^90^Y-1849 with saline or SQAP (2 mg/kg body weight). Tumor sections were stained with hematoxylin and eosin (Sakura Finetek USA, Torrance, CA, USA). Apoptotic cells in tumors were stained by TUNEL staining with a DeadEnd Colorimetric TUNEL system (Promega, Madison, WI, USA). Ki-67 antigen was detected using an anti-human Ki-67 polyclonal antibody (Agilent Technologies Japan, Tokyo, Japan) as described previously [38]. CD31 antigen was detected using an anti-CD31 polyclonal antibody (Abcam, Cambridge, UK) as described previously [23]. Details are provided in the Supplementary information.

### Statistical analysis

All quantitative data are expressed as the means ± standard deviation (SD). The data were analyzed with GraphPad Prism 7 software (GraphPad Software, La Jolla, CA, USA). Details are provided in the Supplementary information.

## Results

### Effect of SQAP on hematologic parameters

To test the pharmacologic effects of SQAP on the blood system after a single-dose administration, hematologic tests were conducted. Compared with the saline-injected control mice, intravenous injection of 2 mg/kg of SQAP had no effect on the number of hemocytes, cell concentrations, hematocrit, or other hematologic parameters tested (Table 1). This trend was also observed in mice injected with a higher dose (up to 32 mg/kg) of SQAP. No noticeable damage to the stomach or tail vein was observed in mice injected with less than 6 mg/kg of SQAP (data not shown). For extra-beam radiotherapy, a 2 mg/kg dose was injected intravenously 20–30 min before irradiation to evaluate effects for sensitizing radiation [32]. This dose was used in the following experiments.

**Table 1:** Hematologic parameters on the SQAP treatment.

SQAP	mg/kg	0 (saline)	2	24	28	32
WBC	10^2^/μl	41.0 ± 11.0	51.0 ± 2.8	102.0 ± 9.2	66.5 ± 21.9	55.0 ± 25.5
RBC	10^4^/μl	769.5 ± 258.0	889.5 ± 156.0	960.6 ± 26.0	923.5 ± 53.0	633.5 ± 319.0
HGB	g/*d*l	10.8 ± 4.7	12.9 ± 2.3	13.7 ± 0.4	13.1 ± 1.0	9.1 ± 4.7
HCT	%	37.2 ± 13.1	41.7 ± 7.2	44.5 ± 11.7	42.6 ± 3.3	30.1 ± 15.4
MCV	*F*l	48.1 ± 0.8	46.9 ± 0.1	46.4 ± 0.5	46.2 ± 0.8	47.5 ± 0.5
MCH	*p*g	13.8 ± 1.4	14.5 ± 0.0	14.2 ± 0.0	14.2 ± 0.3	14.3 ± 0.1
MCHC	g/*d*l	28.7 ± 2.5	31.0 ± 0.1	30.7 ± 0.4	30.8 ± 0.1	30.2 ± 0.0
PLT	10^4^/μl	39.3 ± 19.4	58.9 ± 7.7	60.4 ± 0.0	62.2 ± 5.34	39.2 ± 20.1

WBC: white blood cells, RBC: red blood cells, HGB: hemoglobin content, HCT: hematocrit, MCV: mean cell volume, MCH: mean corpuscular hemoglobin, MCHC: mean cell hemoglobin concentration, PLT: platelet number.

### Biodistribution of combined ^111^In-labeled 1849 and SQAP in mice bearing BxPC-3 tumors

**Fig. 2:**
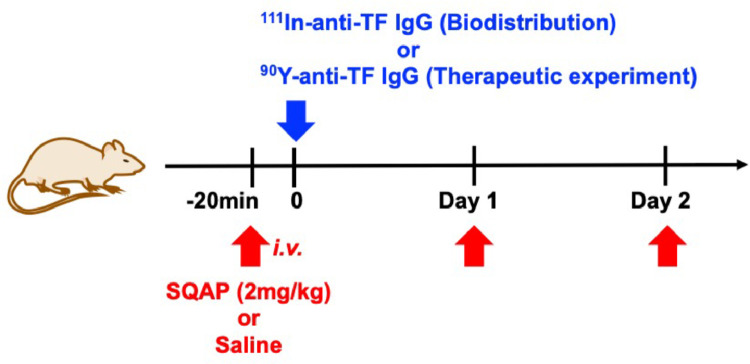
Schema of the study design to evaluate biodistribution (^111^In-labeled 1849) and radioimmunotherapy (^90^Y-labeled 1849) with the radiolabeled anti-tissue factor (TF) antibody 1849 and saline or SQAP.

**Fig. 3:**
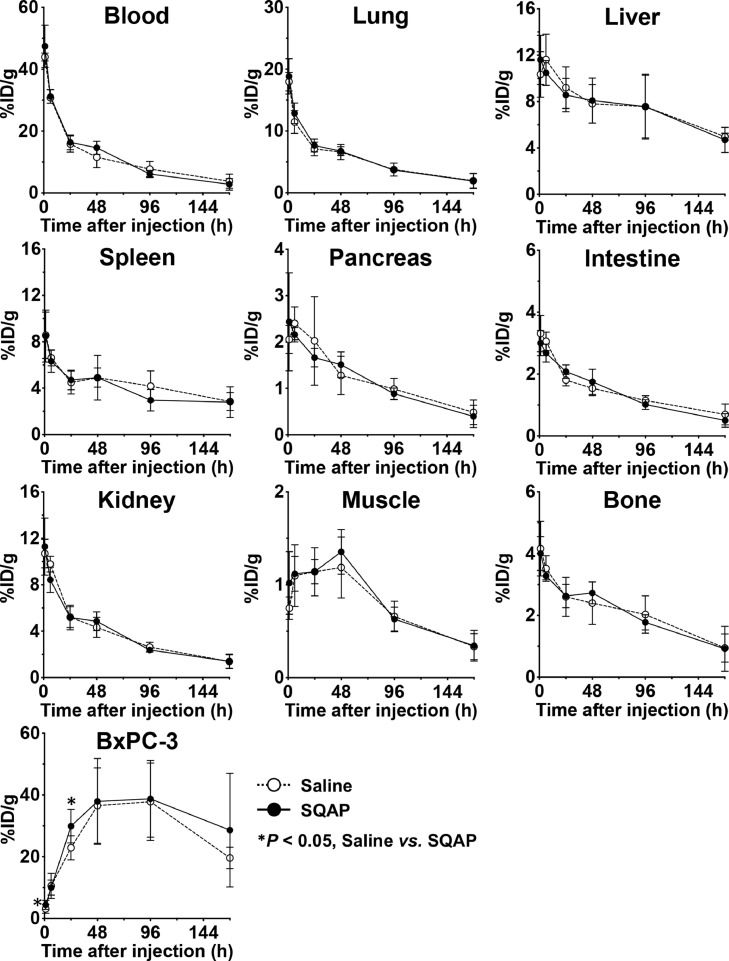
Biodistribution of ^111^In-labeled 1849 with SQAP in nude mice bearing BxPC-3 xenografts. Tracer uptake was quantified at 1, 6, 24, 48, 96, and 168 h after intravenous injection of 37 kBq of ^111^In-labeled 1849 with saline or SQAP (2 mg/kg body weight). Data are expressed as mean ± SD. *P < 0.05.

As the schedule shows in Fig. 2, biodistribution experiments of ^111^In-labeled 1849 were conducted in nude mice bearing BxPC-3 subcutaneous tumors at 1 to 168 h after injection of the antibody (*n* = 5 each time-point). To evaluate the effect of SQAP on biodistribution, the mice were injected intravenously with saline or SQAP at 20 min before ^111^In-1849 injection, and 24 and 48 h after the injection. Tumor uptake of ^111^In-1849 at 1 h after injection was 3.2 ± 1.5% ID/g in the saline-injected group and 4.4 ± 1.4% ID/g in the SQAP-injected group, and uptake increased until 48 h after injection (Fig. 3). The peak values were 37.8 ± 12.5% ID/g in the saline group and 38.8 ± 12.4% ID/g in the SQAP group at 48 h after injection, and these values were almost maintained for up to 96 h after the injection and then decreased (Fig. 3). Tumor uptake differed significantly between the saline and SQAP groups at 24 h after injection (*P* < 0.05; Fig. 3). The time-activity curves in the blood and major organs were similar between groups with no significant difference (Fig. 3). On the basis of these results, the absorbed dose was estimated by replacing ^111^In with the therapeutic radioisotope ^90^Y, as shown in Table 2***.*** The dose absorbed by tumors in the saline group injected with 0.925, 1.85, and 3.7 MBq of ^90^Y-labeled 1849 was estimated to be 10.9, 21.8, and 43.7 Gy, respectively, and that in the SQAP group was 12.0, 23.9, and 47.9 Gy, respectively (Table 2). The absorbed doses to normal organs were similar between the saline and SQAP groups (Table 2).

**Table 2:** Absorbed dose estimation (Gy) for ^90^Y-labeled 1849 in each organ or tissue.

	Saline			SQAP (2 mg/mL)		
	0.925 MBq	1.85 MBq	3.7 MBq	0.925 MBq	1.85 MBq	3.7 MBq
Lung	2.6	5.1	10.3	2.7	5.4	10.8
Liver	3.3	6.5	13.1	3.2	6.4	12.8
Spleen	1.9	3.7	7.5	1.7	3.5	7.0
Pancreas	0.6	1.1	2.3	0.5	1.1	2.2
Intestine	0.7	1.3	2.6	0.7	1.3	2.6
Kidney	1.9	3.7	7.4	1.8	3.6	7.3
Muscle	0.4	0.7	1.5	0.4	0.8	1.5
Bone	0.9	1.9	3.8	0.9	1.9	3.8
BxPC-3	10.9	21.8	43.7	12.0	23.9	47.9

### Treatment with ^90^Y-labeled 1849 and SQAP in mice bearing BxPC-3 tumors

**Fig. 4:**
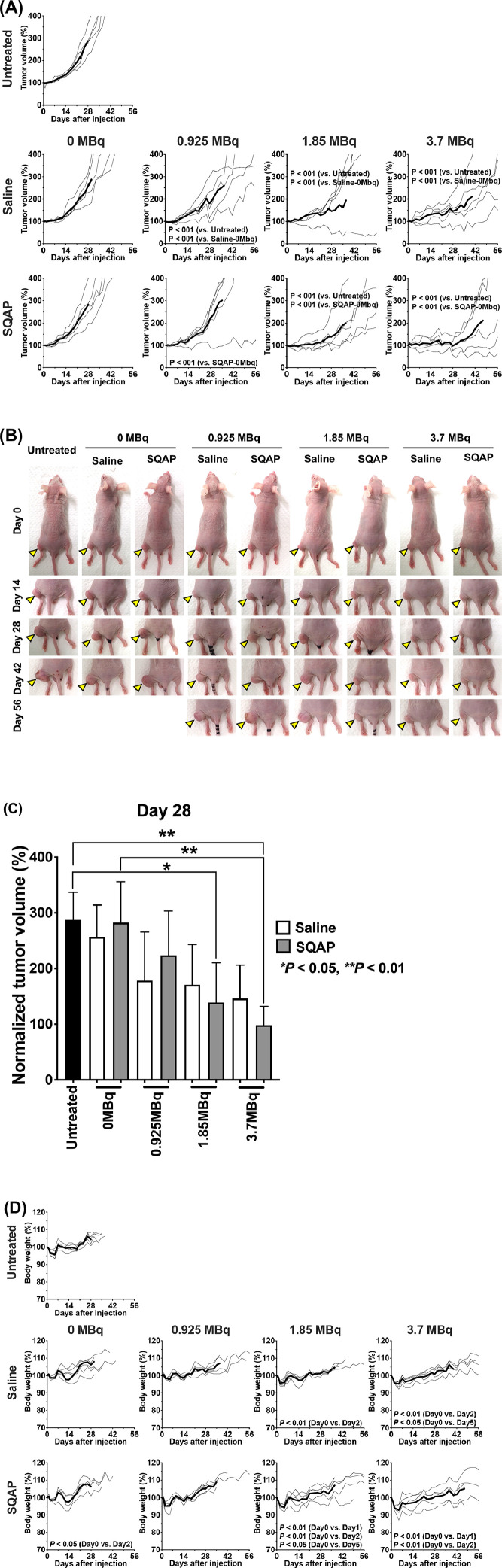
Therapeutic experiments in mice bearing BxPC-3 xenografts. (**A**) Growth curves of BxPC-3 tumors in mice treated with ^90^Y-labeled 1849 and SQAP. Mice were injected intravenously with 0 MBq (unlabeled antibody only), 0.925, 1.85, and 3.7 MBq of ^90^Y-labeled 1849, and saline or SQAP (2 mg/kg body weight). Tumor size was measured at least 3 times a week. Data are presented as mean ± SD. Tumor growth curves of an individual tumor are shown as thin lines and the mean tumor growth curve is shown as a bold line. **(B)** Representative photos of mice at days 0, 14, 28, 42, and 56 after intravenous injection with ^90^Y-labeled 1849 antibody (37 MBq), and saline or SQAP (2 mg/kg body weight). Arrowheads indicate tumors. (**C**) Normalized tumor volumes at day 28 after the treatment. *P < 0.05, **P < 0.01. **(D)** Body weight curves of mice bearing BxPC-3 tumors treated with ^90^Y-labeled 1849 and SQAP. Mice were intravenously injected with 0 MBq (unlabeled antibody only), 0.925, 1.85, and 3.7 MBq of ^90^Y-labeled 1849, and saline or SQAP (2 mg/kg body weight). Body weight was measured at least 3 times a week. Body weight curves of individual mice are shown as thin lines and the mean body weight curve is shown as a bold line. Body weights were compared with those on day 0 and analyzed by repeated measures 1-way ANOVA with Dunnett's multiple comparison test. *P < 0.05, **P < 0.01.

The treatment effects of ^90^Y-labeled 1849 with saline or SQAP were evaluated based on tumor volume and body weight in mice bearing BxPC-3 tumors (Fig. 4). Tumor growth curves are shown in Fig. 4**A** and representative mouse photos are shown in Fig. 4**B**. No statistically significant difference was observed in the 3 control groups (Fig. 4**A**). By contrast, treatment with ^90^Y-1849 (0.925, 1.85, and 3.7 MBq) significantly suppressed tumor growth compared with no treatment, or treatment with saline or SQAP alone (*P* < 0.01; Fig. 4**A** and 4**B**). Treatment with 3.7 MBq ^90^Y-1849 with SQAP had the greatest tumor suppression effect: BxPC-3 tumor growth was suppressed until around 28 days after treatment (Fig. 4**A,** 4**B,** and 4**C**), and tumor volumes gradually increased thereafter (Fig. 4**A**). In 2 of the 5 mice treated with 3.7 MBq ^90^Y-1849 with SQAP, tumor growth suppression continued to around day 56 (Fig. 4**A** and 4**B**). The therapeutic effect of 1.85 MBq ^90^Y-1849 with SQAP on tumor growth suppression was comparable to that of 3.7 MBq ^90^Y-1849 with saline (Fig. 4**C**). Survival was most prolonged in mice treated with 3.7 MBq of ^90^Y-1849 with SQAP: 100% until day 33 and then decreasing to 40% at day 56 (end of the observation period), followed by 20% at day 56 in the groups receiving 0.925 MBq of ^90^Y-1849 with SQAP, 1.85 MBq of ^90^Y-1849 with saline and SQAP, and 3.7 MBq of ^90^Y-1849 with saline (**Supplementary Figure 1**). Fig. 4**D** shows temporal body weight changes in mice. Compared with day 0, significant transient body weight loss was observed in the following treatments groups: SQAP alone (*P* < 0.05), 1.85 MBq of ^90^Y-1849 with saline (*P* < 0.01) and SQAP (*P* < 0.01 and *P* < 0.05), and 3.7MBq of ^90^Y-1849 with saline (*P* < 0.01 and *P* < 0.05) and SQAP (*P* < 0.01). The decreased body weight recovered within several days (Fig. 4**D**). No visible adverse effects, such as diarrhea and dyspnea, were observed at any dose level.

### Histologic analysis of BxPC-3 tumors treated with ^90^Y-labeled 1849 and SQAP

**Fig. 5:**
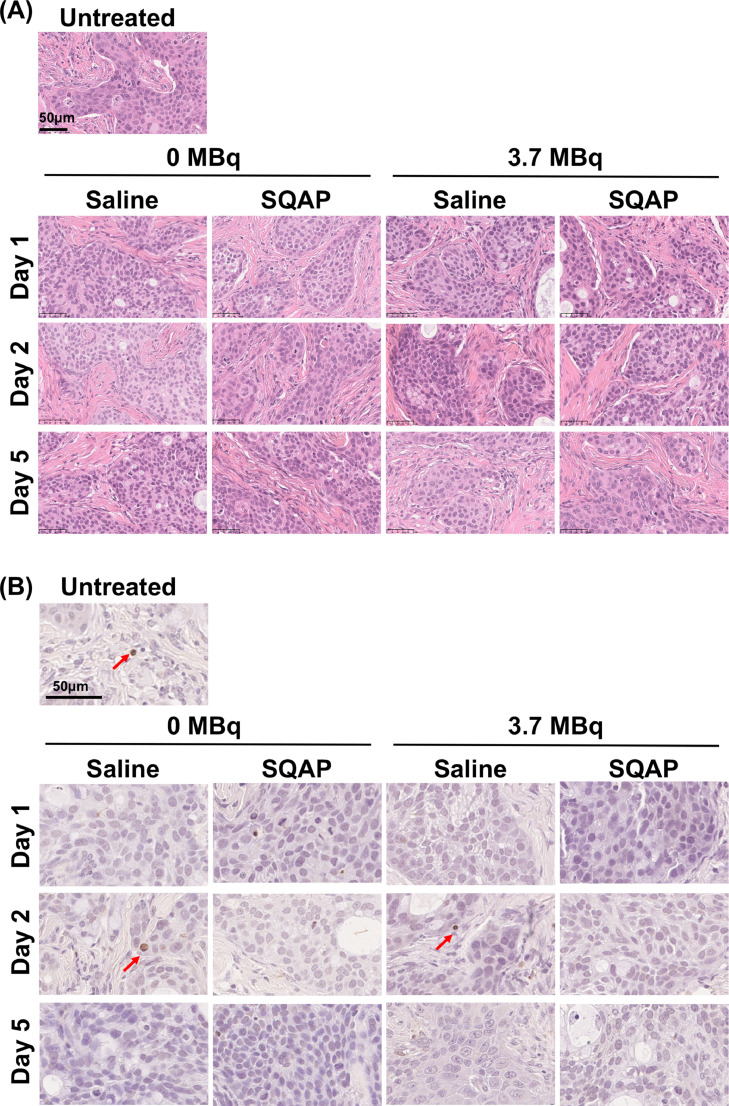
Histologic analysis of BxPC-3 tumors treated with ^90^Y-labeled 1849 and SQAP. Mice were injected intravenously with 0 MBq (unlabeled antibody only) and 3.7 MBq of ^90^Y-labeled 1849, and saline or SQAP (2 mg/kg body weight). Tumors were sampled at 1, 2, and 5 days after injection of ^90^Y-1849. (**A**) Hematoxylin-and-eosin-stained sections. (**B**) TUNEL-stained sections.

Histologic changes were evaluated in groups of mice treated with unlabeled 1849 (0 MBq) and saline or SQAP, or with 3.7 MBq of ^90^Y-labeled 1849 and saline or SQAP. Hematoxylin and eosin-stained sections of BxPC-3 tumors did not exhibit marked differences in any treatment groups compared with the untreated group as a control (Fig. 5**A**). As shown in Fig. 5**B**, TUNEL-stained sections showed few apoptotic cells in any treatment groups.

**Fig. 6:**
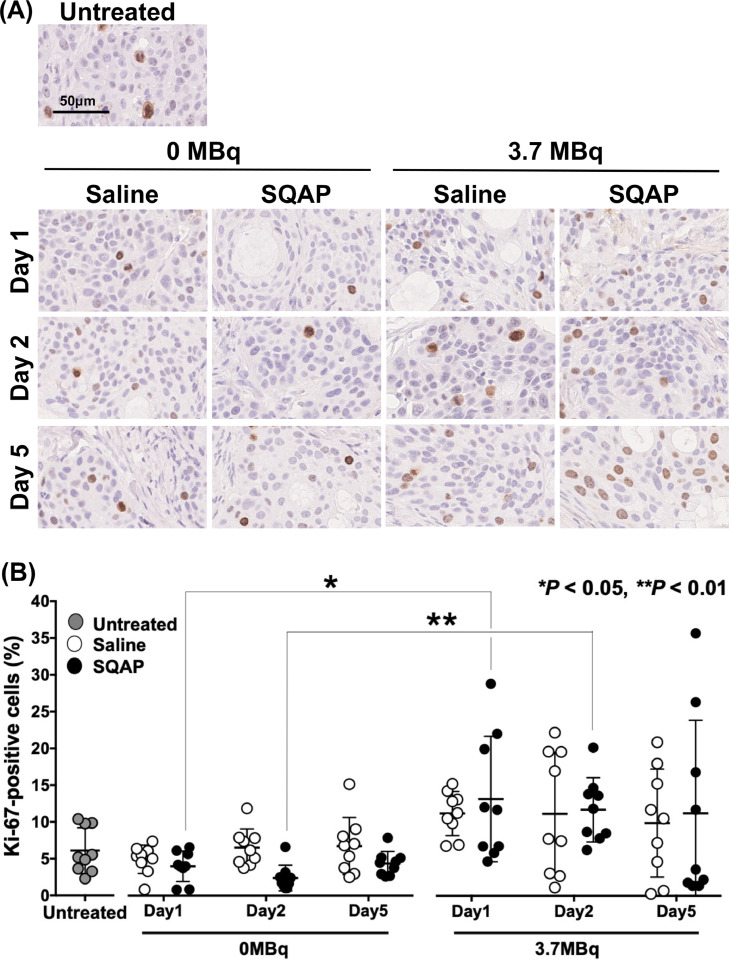
Histologic analysis of Ki-67-positive proliferation cells. (**A**) Ki-67-stained sections of BxPC-3 tumors treated with ^90^Y-labeled 1849 and SQAP. Mice were injected intravenously with 0 MBq (unlabeled antibody only) and 3.7 MBq of ^90^Y-labeled 1849, and saline or SQAP (2 mg/kg body weight). Tumors were sampled at 1, 2, and 5 days after injection of ^90^Y-1849. The Ki-67 antigen was detected using an anti-human Ki-67 polyclonal antibody as the primary antibody (diluted 1:100). (**B**) Scatter plots of positive cells in tumors treated with ^90^Y-labeled 1849, and saline (white circles) or SQAP (black circles). Bars indicate the mean ± SD. *P < 0.05, **P < 0.01.

To evaluate the effect on cell proliferation, Ki-67 staining was conducted (Fig. 6**A**) and the positive cells were quantified (Fig. 6**B**). There was no significant difference between saline and SQAP treatments in any antibody treatments and on any days (Fig. 6**A** and 6**B**). Significant differences were observed between mice treated with 0 MBq and 3.7 MBq ^90^Y-1849: 3.7 MBq with SQAP increased the number of Ki-67-positive swollen cells compared with 0 MBq with SQAP at day 1 (*P* < 0.05) and day 2 (*P* < 0.01) as shown in Fig. 6**B**. Although there was no statistical difference, treatment using 3.7 MBq ^90^Y-1849 with saline tended to increase the number of Ki-67-positive cells (Fig. 6**B**).

**Fig. 7:**
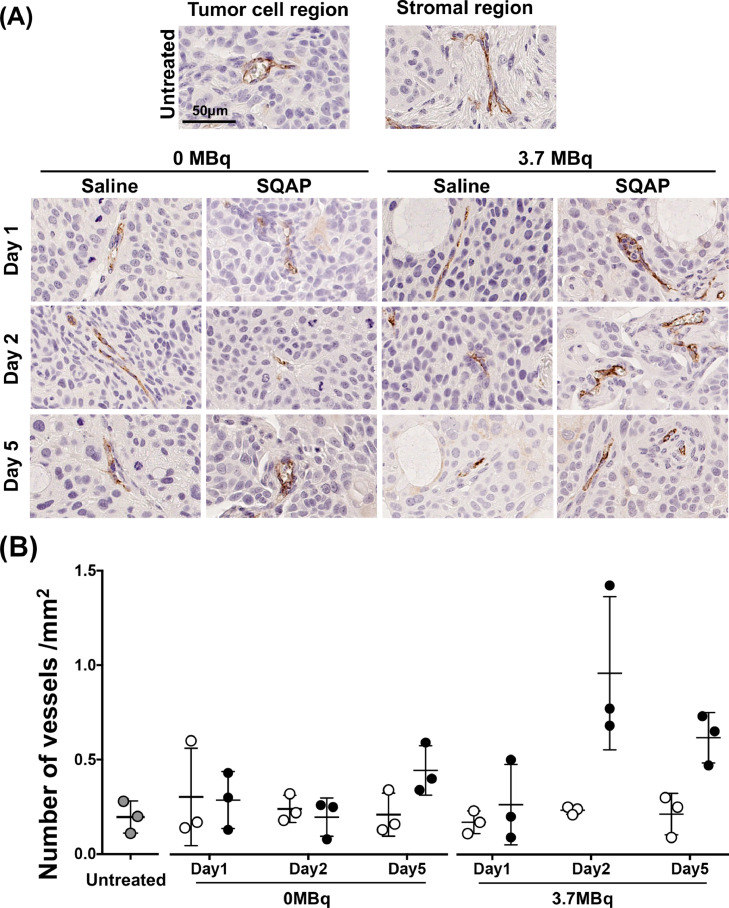
Histologic analysis of CD31-positive proliferation cells. (**A**) Representative CD31-stained sections of BxPC-3 tumors. The tumors were sampled at days 1, 2, and 5 after intravenous injection of ^90^Y-labeled 1849 with or without SQAP. Mice were injected intravenously with 0 MBq (unlabeled antibody only) and 3.7 MBq of ^90^Y-labeled 1849, and saline or SQAP (2 mg/kg body weight). The antigens were detected using an anti-CD31 polyclonal antibody as the primary antibody (diluted 1:50). (**B**) Scatter plots of positive cells in tumors treated with ^90^Y-labeled 1849, and saline (white circles) or SQAP (black circles). There was no significant difference.

To evaluate the blood vessels in tumors after the treatments, tumors were stained with the endothelial cell marker CD31. Most vessels were observed in stromal tissues but some were found in tumor cell regions of untreated BxPC-3 tumors (Fig. 7**A** and 7**B**). Although the number of vessels was markedly increased at day 2 in the group treated using 3.7 MBq ^90^Y-1849 with SQAP, the difference was not statistically significant (Fig. 7**A** and 7**B**).

## Discussion

The findings of the present study revealed that SQAP enhanced the efficacy of RIT with ^90^Y-labeled 1849 against BxPC-3 tumors. This is likely because SQAP enhanced the tumor uptake of radiolabeled antibodies by increasing tumor perfusion, as observed on photoacoustic imaging and dynamic contrast-enhanced magnetic resonance imaging of tumors [32]. Radiolabeled 1849 showed high uptake in BxPC-3 by active targeting in addition to passive targeting (i.e., enhanced permeability and retention effect; EPR [39]). SQAP also increases tumor oxygenation, which can enhance the cell-killing effects of radiation [32]. These functions of SQAP would enhance the antitumor effect of ^90^Y-labeled 1849, further suppressing tumor growth beyond that of ^90^Y-1849 alone by increasing the absorbed dose from 43.7 to 47.9 Gy when injected with 3.7 MBq. In addition to increased perfusion, enhanced tumor oxygenation may play an important role in creating a radiosensitizing effect [32]. Noteworthy, 1.85 MBq of ^90^Y-1849 with SQAP provided an antitumor effect equivalent to 3.7 MBq of ^90^Y-1849 without SQAP; hence, SQAP administration can decrease the injected dose of radiotherapeutic agents to half. That means radiation doses absorbed by organs and tissues are reduced by half, suggesting that SQAP would contribute to reducing radiologic toxicity in patients. Our findings suggest that SQAP is a potentially powerful tool to enhance the efficacy of RIT with lower toxicity, and TF-targeted RIT with SQAP is a promising therapeutic option for pancreatic cancer, even those that have metastasized.

The increased perfusion and oxygenation induced by the addition of SQAP continue for only a short period [32]. Nevertheless, the enhanced antitumor effects were observed for a long period in the present study. Brief effects on perfusion were supported in the present study: the largest difference in the tumor uptake of the radiolabeled antibody was detected at 1 h (1.4-fold tumor uptake), and thereafter no significant difference was detected. Tumor growth suppression was observed for 28 days, although SQAP injection terminated on day 2. Tumor vascular formation increased in tumor cell regions treated with ^90^Y-1849, especially in treatment groups also treated with SQAP. SQAP itself has no angiogenetic effects, thus the increased tumor uptake of ^90^Y-1849 by SQAP enhances the absorbed radiation dose and cognate vascular formation, as is the case with neoangiogenesis induced by external-beam radiation [40]. This causes a wider intratumoral biodistribution of ^90^Y-1849, enhancing tumor cell damage. Indeed, our histologic analysis revealed an increase in the number of Ki-67-positive cells, which was reported as an index after external-beam radiation [41], providing evidence for increased irradiation to BxPC-3 cells. Both increased tumor uptake by SQAP and enhanced vascular formation at early time-points could contribute to increase radiation exposure to tumors, resulting in tumor cell damage for a long period. Further investigations are necessary to better understand the mechanism of SQAP-induced radiosensitization in RIT.

SQAP enhanced the efficacy of RIT; unfortunately, the combination therapy did not lead to complete remission. Additional strategies are needed to improve the efficacy. First, SQAP dose escalation may further enhance treatment. In the present study, we used a low dose of 2 mg/kg for combination therapy, but higher doses from 4 to 18 mg/kg were tolerable in mice. Higher doses would enhance perfusion and oxygenation, perhaps conferring higher antitumor effects. Further investigation to determine the optimal dose and schedule of SQAP is required to maximize the efficacy of the combination therapy with few side effects. Second, a new SQAP derivative with long blood circulation could be an option. External-beam radiotherapy includes short-time radiation with a high dose rate, while RIT includes continuous radiation with a low dose rate. The SQAP effects do not continue for a long period [32], and SQAP was injected 3 times for a single shot of ^90^Y-1849. The short-term SQAP effects are due to its short half-life in the blood (unpublished data). Extending the half-life might enhance the efficacy of RIT. Because a long half-life could increase hematologic toxicity, it is necessary to consider the balance of toxicity and benefit.

The present study has several limitations. First, although SQAP improved the efficacy of RIT, complete remission was not achieved. Further studies are needed to optimize the dose and schedule for RIT, and develop a new derivative with a long half-life suitable for RIT, as mentioned above. Second, RIT can target metastatic cancer, but the present study includes only a subcutaneous tumor model. Most pancreatic cancers are diagnosed at advanced stages with invasive and metastatic cancer cells. Our TF-targeted RIT with SQAP has the potential to treat such cancer cells. It is necessary to evaluate the efficacy in metastatic models. Such investigations will increase the potential benefit of SQAP in RIT to provide better outcomes.

## Conclusion

The findings of the present study indicated that SQAP enhanced the antitumor effect of RIT as well as external-beam radiotherapy. Administration of SQAP improved the efficacy of RIT with ^90^Y-labeled 1849 in the stroma-rich pancreatic cancer model BxPC-3 by increasing tumor uptake and cognate biologic actions in the early phase of the treatment. Combining ^90^Y-labeled 1849 with SQAP produced a similar antitumor effect with only a half dose of ^90^Y-labeled 1849 without increasing adverse effects. The use of SQAP for clinical RIT is a promising therapeutic option for malignant pancreatic cancer. Because high TF expression is observed in many types of cancer other than pancreatic cancer [9, 10, 42], SQAP could be applicable to enhance the therapeutic efficacy of TF-targeted RIT to different cancers. Our findings warrant further investigations for clinical application.

## Author contributions

Conception/design: Y.T., A.B.T.

Investigation: Y.T., A.S., A.B.T.

Methodology: A.S., A.B.T., H.S., M.Y., Y.M.

Manuscript preparation: Y.T., A.S., A.B.T.

Technical or material support: A.S. A.B.T., H.S., M.Y., Y.M., F.S., K.S.

Study supervision: A.B.T., T.H.

## Declaration of competing interest

Fumio Sugawara and Kengo Sakaguchi are employees of MT3 Technologies Inc. The other authors have no financial or other competing interests to declare in relation to this study.

## Appendix Supplementary materials

Image, application 1

Image, application 2
