# Supplementary material for: The natural sulfoglycolipid derivative SQAP improves the therapeutic efficacy of tissue factor-targeted radioimmunotherapy in the stroma-rich pancreatic cancer model BxPC-3
Source: Transl Oncol. 2021 Nov 25;15(1):101285. doi: 10.1016/j.tranon.2021.101285 (PMC8628266; doi:10.1016/j.tranon.2021.101285)
Supplement: Supplementary file 1 [file mmc1.docx]

# Supplementary information

**The natural sulfoglycolipid derivative SQAP improves the therapeutic efficacy of tissue factor-targeted radioimmunotherapy in the stroma-rich pancreatic cancer model BxPC-3**

Yoichi Takakusagi, Aya Sugyo, Atsushi B. Tsuji, Hitomi Sudo, Masahiro Yasunaga, Yasuhiro Matsumura, Fumio Sugawara, Kengo Sakaguchi, and Tatsuya Higashi

# Supplementary materials and methods

***Measurement of the hematological parameters***

The Animal Care and Use Committee of the National Institute of Radiological Sciences approved the protocol for the animal experiments, and all animal experiments were conducted by following the Institutional Guidelines regarding Animal Care and Handling. Male BALB/c-nu/nu mice (5 weeks old, CLEA Japan, Tokyo, Japan) were maintained under specific pathogen-free conditions. SQAP (Toyo Suisan, Tokyo, Japan) was dissolved in saline (Otsuka Pharmaceutical, Tokyo, Japan). Mice (n = 2) were intravenously administered SQAP (2, 24, 28 and 32 mg/kg body weight) or saline. Blood was collected *via* the tail vein at day 1, and the white blood cells, red blood cells, hemoglobin, hematocrit, mean corpuscular volume, mean corpuscular hemoglobin, mean corpuscular hemoglobin concentration, and platelets were immediately analyzed with a Celltac Alpha hematology analyzer (Nihon Kohden, Tokyo, Japan).

***Radiolabeling of antibody***

An anti-tissue factor (TF) monoclonal antibody 1849 was conjugated with *p*-SCN-Bn-CHX-A''-DTPA (DTPA; Macrocyclics, Dallas, TX, USA) as previously described [1]. The DTPA-conjugated antibody was purified using a Sephadex G-50 (GE Healthcare, Little Chalfont, UK) column (700 × g for 2 min). The conjugation ratio of DTPA to antibody was estimated to be approximately 1.5 by cellulose acetate electrophoresis. For In-111 labeling, 125 μg of DTPA-conjugated antibody was mixed with 1.85 MBq ^111^InCl_3_ in 0.5 M acetate buffer (pH 6.0), and the mixture was incubated for 30 min at room temperature. For Y-90 labeling (two experiments), 150 μg of DTPA-conjugated antibody was mixed with 111 MBq ^90^YCl_3_ in 0.5 M acetate buffer (pH 6.0), and the mixture was incubated for 30 min at room temperature. The radiolabeled antibodies were separated from free radionuclides on a Sephadex G-50 column (700 × g for 2 min).

Histologic analysis of BxPC-3 tumors treated with ^90^Y-labeled 1849 and SQAP

As a separate experiment, tumor samples (n = 3/time-point) were extirpated at days 1, 2, and 5 after intravenous injection of intact 1849 (defined as 0 MBq) or 3.7 MBq of ^90^Y-1849 with saline or SQAP (2 mg/kg body weight). Untreated tumors (n = 3) were used as a control. The tumors were fixed in 10% (v/v) neutral buffered formalin and embedded in paraffin for sectioning. Sections (5-μm thick) were stained with hematoxylin and eosin (Sakura Finetek USA, Torrance, CA, USA). Apoptotic cells in tumors were stained by terminal deoxynucleotidyl transferase-mediated deoxyuridine triphosphate nick-end labeling (TUNEL) staining with a DeadEnd Colorimetric TUNEL system (Promega, Madison, WI, USA). TUNEL-positive cells were quantified by counting three randomly selected fields of each section at 400× magnification. Ki-67 antigen was detected using an anti-human Ki-67 polyclonal antibody (Agilent Technologies Japan, Tokyo, Japan) as described previously [2]. Ki-67-positive cells were quantified by counting three randomly selected fields of each section at 400× magnification. CD31 antigen was detected using an anti-CD31 polyclonal antibody (Abcam, Cambridge, UK) as described previously [1]. CD31-positive vascular endothelial cells only in the tumor cell area (not in the stromal area) were counted in three randomly selected fields of each section at 400× magnification.

Statistical analysis

All quantitative data are expressed as the means ± standard deviation (SD). The biodistribution data were analyzed by Mann-Whitney test using GraphPad Prism 7 software (GraphPad Software, La Jolla, CA, USA). The tumor volume and body weight data were normalized by the measurements at day 0. The tumor volume data were analyzed between treatment groups by two-way ANOVA using GraphPad Prism 7 software. The body weight data after treatments were compared with the data at day 0 by one-way ANOVA with Dunnett's multiple comparison test using GraphPad Prism 7 software. The histologic analysis data were analyzed by one-way ANOVA with Dunnett's multiple comparison test using GraphPad Prism 7 software (GraphPad Software, La Jolla, CA, USA).

# Supplementary references

[1] A. Sugyo, A.B. Tsuji, H. Sudo, M. Okada, M. Koizumi, H. Satoh, G. Kurosawa, Y. Kurosawa, T. Saga, Evaluation of Efficacy of Radioimmunotherapy with 90Y-Labeled Fully Human Anti-Transferrin Receptor Monoclonal Antibody in Pancreatic Cancer Mouse Models, PLoS One, 10 (2015) e0123761.

[2] H. Sudo, A.B. Tsuji, A. Sugyo, Y. Ogawa, M. Sagara, T. Saga, ZDHHC8 knockdown enhances radiosensitivity and suppresses tumor growth in a mesothelioma mouse model, Cancer Sci, 103 (2012) 203-209.

# Supplementary Figure

**Supplementary Figure 1.** Survival curves of BxPC-3-tumor-bearing mice treated with ^90^Y-labeled 1849 and SQAP. Mice were intravenously injected with 0 (unlabeled antibody only), 0.925, 1.85, and 3.7 MBq of ^90^Y-labeled 1849, and saline or SQAP (2 mg/kg body weight). Tumor size was measured at least three times a week. Survival curve based on the endpoint of 200% tumor volume.
