# Supplementary material for: The natural sulfoglycolipid derivative SQAP improves the therapeutic efficacy of tissue factor-targeted radioimmunotherapy in the stroma-rich pancreatic cancer model BxPC-3
Source: Transl Oncol. 2021 Nov 25;15(1):101285. doi: 10.1016/j.tranon.2021.101285 (PMC8628266; doi:10.1016/j.tranon.2021.101285)
Supplement: Supplementary file 2 [file mmc2.pdf]

SciTechEdit INTERNATIONAL

Communicate Your Science Clearly

English-Language Science Editing

16 July 2021

This letter certifies that SciTechEdit International, LLC copy-edited a version (July, 2021) of the manuscript originally titled **“The natural sulfoglycolipid derivative SQAP improves the therapeutic efficacy of tissue factor-targeted radioimmunotherapy in the stroma-rich pancreatic cancer model BxPC-3”** by Y. Takakusagi, A. Sugyo, A.B. Tsuji, H. Sudo, M. Yasunaga, Y. Matsumura, F. Sugawara, K. Sakaguchi, and T. Higashi[1].

SciTechEdit International takes full responsibility for the processing and correction of documents while in our hands. Implementation of the editorial changes suggested by SciTechEdit International, LLC and changes made after the edited manuscript was returned to the authors are at the sole discretion of the authors.

Please direct any questions regarding the original editing of this manuscript to me at the email address below.

Sincerely,

Karin Mesches, PhD
President, SciTechEdit International
karin.mesches@scitechedit.com

SciTechEdit *International*, *LLC* • 7012 E. Mountain Brush Circle, Highlands Ranch, CO 80130 • Fax: 1.303.773.6660 • SciTechEdit.com
